# Absence of *VHL* gene alteration and high VEGF expression are associated with tumour aggressiveness and poor survival of renal-cell carcinoma

**DOI:** 10.1038/sj.bjc.6605298

**Published:** 2009-09-15

**Authors:** J-J Patard, N Rioux-Leclercq, D Masson, S Zerrouki, F Jouan, N Collet, C Dubourg, B Lobel, M Denis, P Fergelot

**Affiliations:** 1 Department of Urology, Rennes University Hospital, Rennes, France; 2 Department of Pathology, Rennes University Hospital, Rennes, France; 3 CNRS UMR 6061, Institut de Génétique et Développement, University Rennes 1, Rennes, France; 4 INSERM U913, Institut des Maladies de l’Appareil Digestif, Nantes University Hospital, Nantes, France; 5 Department of biochemistry and molecular genetics, Rennes University Hospital, Rennes, France

**Keywords:** renal-cell carcinoma, VHL, VEGF

## Abstract

**Background:**

The von Hippel–Lindau gene (*VHL*) alteration, a common event in sporadic clear-cell renal-cell carcinoma (CCRCC), leads to highly vascularised tumours. Vascular endothelial growth factor (VEGF) is the major factor involved in angiogenesis, but the prognostic significance of both *VHL* inactivation and VEGF expression remain controversial. The aims of this study were to analyse the relationship between *VHL* genetic and epigenetic alterations, VHL expression and VEGF tumour or plasma expression, and to analyse their respective prognostic value in patients with CCRCC.

**Methods:**

A total of 102 patients with CCRCC were prospectively analysed. Alterations in *VHL* were determined by sequencing, Multiplex Ligation-dependent Probe Amplification (MLPA) and methylation-specific MLPA. Expression of pVHL and VEGF was determined by immunohistochemistry. Plasma VEGF was measured by enzyme-linked immunosorbent assay (ELISA).

**Results:**

*VHL* mutation, deletion and promoter methylation were identified in 70, 76 and 14 cases, respectively. Overall, at least one *VHL*-gene alteration occurred in 91 cases (89.2%). Both VEGF tumour and plasma expression appeared to be decreased in case of *VHL* alteration. Median progression-free survival and CCRCC-specific survival were significantly reduced in patients with wild-type *VHL* or altered *VHL* and high VEGF expression, which, therefore, represent two markers of tumour aggressiveness in CCRCC.

**Conclusion:**

Stratifying CCRCCs according to VHL and VEGF status may help tailor therapeutic strategy.

Renal-cell carcinoma (RCC) accounts for 3% of all solid tumours. Seventy-five to 85% of RCCs are clear-cell carcinomas (clear-cell renal-cell carcinoma (CCRCC)), which are highly vascularised tumours. As much as 30% of patients do have metastases at the time of diagnosis and an additional 30–40% will develop metastases during follow-up, although a radical surgery has been initially performed (Rabinovitch *et al*, 1994). Significant progresses have recently been made in the medical treatment of metastatic RCC by targeting a number of growth factors, including vascular endothelial growth factor (VEGF) and platelet-derived growth factor (PDGF) and their receptors (Motzer and Bukowski, 2006; Patard *et al*, 2008a). Indeed, median progression-free survival (PFS) time has been doubled either in first-line or in second-line therapy by targeting tumour angiogenesis (Motzer *et al*, 2007).

The von Hippel–Lindau (*VHL*) tumour suppressor has been isolated in 1993 (Latif *et al*, 1993). The protein encoded by the *VHL* gene (pVHL) is the substrate recognition component of a ubiquitin ligase complex that targets a transcription factor, hypoxia-inducible factor (HIF), for proteolysis. A biallelic *VHL* inactivation leads to HIF-1*α* accumulation and subsequent overexpression of genes, which are critical for tumour angiogenesis, cell proliferation and migration (Knebelmann *et al*, 1998; Cockman *et al*, 2000; Kim and Kaelin, 2004).

Although some studies have shown a positive relationship between genetic and epigenetic *VHL* gene alterations and VEGF tumour overexpression (Igarashi *et al*, 2002; Na *et al*, 2003), literature results are conflicting especially regarding the association between *VHL* mutation, usual prognostic parameters and survival (van Houwelingen *et al*, 2005; Schraml *et al*, 2002; Yao *et al*, 2002). In a recent study we have demonstrated that the absence of *VHL* mutation, associated with low Carbonic Anhydrase IX (CAIX) tumour expression, was associated to a particular aggressive CCRCC phenotype (Patard *et al*, 2008b). Both CAIX and VEGF are VHL/HIF downstream targets, but unlike CAIX, which is a surrogate indicator for VHL status, VEGF regulation is much more complex. Conflicting hypotheses also exist regarding its prognostic value in CCRCC (Jacobsen *et al*, 2002). Therefore, we aimed this prospective study for evaluating the association between *VHL* statu*s* (mutation, deletion, promoter methylation and pVHL expression), tumour VEGF expression, plasma VEGF levels and usual prognostic parameters in CCRCC. We subsequently analysed the respective prognostic value of these different biological tumour characteristics.

## Materials and methods

### Study population

A total of 102 patients operated for a sporadic CCRCC at the Department of Urology at the Rennes University Hospital between 2003 and 2004 were analysed. In case of distant metastases, patients were treated following surgery according to the standard of care of the period, which was immunotherapy. Surveillance following surgery included repeated clinical assessments, blood biochemistry tests, chest and abdominal CT scans. No patient was lost to follow-up. The study protocol was approved by the institutional ethics committee and informed consent for participating in this study was obtained in each case. The clinicopathological data of the patients are summarised in Table 1.

### Pathological analysis

Formalin-fixed paraffin sections were stained with hematoxylin and eosin–safran for light microscopy. The slides were reviewed by one pathologist (N Rioux-Leclercq). Only conventional CCRCCs were considered for analysis. Macroscopic and histological parameters, which were analysed included tumour size, tumour necrosis and nuclear Fuhrman grade (Fuhrman *et al*, 1982; Rioux-Leclercq *et al*, 2007b). Tumour stage was defined according to the TNM classification (Guinan *et al*, 1997).

### Tissue sample management

All consecutive conventional CCRCCs and paired renal cortex samples from untreated patients undergoing partial or total nephrectomy were analysed. Immediately after macroscopic examination, small samples were collected from surgical specimens, frozen in liquid nitrogen and stored at –80°C. Genomic DNA was extracted using QIAamp DNA minikit (Qiagen, Courtaboeuf, France).

### VEGF plasma measurement

Blood samples were collected before nephrectomy and processed as described (Rioux-Leclercq *et al*, 2007a). The plasma was stored at −80°C until analysed. Quantikine human VEGF Immunoassay (R&D Systems Europe, Lille, France) enzyme-linked immunosorbent assay (ELISA) was performed to quantitate VEGF levels (VEGF_121_ and VEGF_165_ free isoforms) in plasma. All samples were assessed in duplicate. The value of plasma VEGF (pVEGF) was expressed in pg/ml. The detection limit of the test was 9 pg^−1^ml and the upper limits of normal value used for VEGF in plasma was 62.5 pg ml^−1^ (Adams *et al*, 2000).

### VEGF and pVHL tumour immunostaining

In each case, a representative slide of the tumour with the highest nuclear Fuhrman grade and the corresponding paraffin block were selected for immunohistochemistry. Tissue sections were deparaffinised in xylene using standard procedures.

For VEGF immunostaining, antigen retrieval was performed by immersing sections in a 0.01 M sodium citrate buffer (pH 6.0) and heated in a 600 W microwave four times for 2 min. The slides were then left to cool down for 30 min and rinsed in a phosphate-buffered saline solution. Slides were incubated at room temperature for 1 h with the polyclonal anti-VEGF antibody (sc-152, dilution 1/100; Santa Cruz Biotechnology, Santa Cruz, CA, USA). A biotin–streptavidin detection system (Dako, Glostrup, Denmark) was then used with diaminobenzidine as the chromogen (Sigma-Aldrich, Lyon, France). Negative controls were performed by omitting the primary antibody and by incubating primary antibody with immunising VEGF peptide prior to slide treatment. VEGF immunoreactivity was expressed as the percentage of the VEGF-positive cells by scoring at least 1000 tumour cells at × 400 magnification. The immunohistochemical signals in the CCRCCs were evaluated as membranous and/or cytoplasmic. For prognostic analysis, a 30% cut-off was used according to previous reports (Jacobsen *et al*, 2004).

pVHL immunohistohemical staining of tissue sections was performed with a monoclonal anti-pVHL antibody (Ig33; Neomarkers, Fremont, CA, USA; Schraml *et al*, 2003; Valle *et al*, 2005), in an automated stainer using the ultraview DAB detection kit (Benchmark; Ventana, Tucson, AZ, USA) following antigen retrieval. Semi-quantitative assessment of the antibody staining was performed by a single uropathologist blinded to the clinicopathological variables (N Rioux-Leclercq). A negative control consisted of omitting the primary antibody. As control for loss of pVHL expression, CCRCC tissue from confirmed *VHL* genetic disease was used. The extent of staining was recorded as a percentage of the tumour tissue sample with positive pVHL expression. Tumours expressing pVHL were scored pVHL+, independently of the percentage of stained cells.

### *VHL* mutational analysis

We amplified two overlapping fragments for exon 1 (1A and 1B) and one fragment for each of exons 2 and 3, covering part of the *VHL* 5′UTR, the entire coding sequence and exon–intron junctions (*VHL* GenBank accession no. AF010238). Primer pairs and PCR-sequencing conditions are described elsewhere (Patard *et al*, 2008b). Denaturing high-performance liquid chromatography (DHPLC) screening was carried out on a WAVE Nucleic Acid Fragment Analysis system (Transgenomic, Glasgow, UK) with a DNAsep column. Aberrant peaks were further analysed by direct sequencing using standard procedures. All mutations were confirmed in a second PCR and sequencing reaction.

### *VHL* deletion analysis

Multiplex Ligation-dependent Probe Amplification (MLPA) analysis was used to detect deletions or duplications in the *VHL* gene (Schouten *et al*, 2002). The SALSA MLPA P016B *VHL* probe kit (MRC-Holland, Amsterdam, the Netherlands) was used. The kit contains eight probes to the *VHL* gene (four in exon 1, two in exon 2 and two in exon 3), additional probes to other genes on 3p and control probes to regions telomeric and centromeric from *VHL*. Detailed information on probe sequences, gene loci and chromosome locations can be found at www.mlpa.com.

Genomic DNA (50–200 ng) was denatured and the probes were allowed to hybridise (16 h at 60°C). PCR was performed on the samples in a volume of 50 *μ*l containing 10 *μ*l of the ligation reaction mixture using the PTC 200 thermal cycler (MJ Research, Waltham, MA, USA). Aliquots of 1.5 *μ*l of the PCR reaction were combined with 0.3 *μ*l ROX-labelled internal size standard (Applied Biosystems, Foster City, CA, USA) and 9 *μ*l deionised formamide. Fragments were separated by electrophoresis on an Applied 3130XL capillary sequencer and quantified using the GeneMarker version 1.6 software (SoftGenetics). For copy-number detection, normal control DNA samples were included in each set of MLPA experiments. Interpretation was based on the comparison of peak heights between the control DNA and the tumour sample. Cut-off levels for loss of relative copy number were set at 0.75.

### *VHL* promoter methylation analysis

Methylation-Specific-MLPA (MS-MLPA) was used to detect CpG island methylation (Jeuken *et al*, 2007). The probe design is similar to an ordinary MLPA probe, except that, for the methylation-specific probes, the sequences detected contain a methylation-sensitive restriction site (*Hha*I). The SALSA MS-MLPA kit ME001B Tumour suppressor-1 allows to detect aberrant methylation of CpG islands located in the promoter region of the *VHL* gene. DNA (50–200 ng) was denatured and the probes were allowed to hybridise (16 h at 60°C). The VHL probes used in this study for methylation quantification analysis contained one *Hha*I restriction site in the target recognition sequence. Following hybridisation, the samples were divided into two and one half of the samples was ligated, whereas for the other part of the sample ligation was combined with *Hha*I digestion enzyme. This digestion resulted in ligation of only the methylated sequences. PCR was performed on both parts of the samples and analysed by electrophoresis as described above. Reference unmethylated DNAs, isolated from blood from healthy volunteers, were included in each set of MLPA experiments. The unmethylated DNA will not generate a signal, and a normal probe signal will be detected if the site is methylated.

### Statistical methods

*χ*^2^-test and Student's *t*-test were used for comparing qualitative and quantitative variables, respectively. Kaplan–Meier method and log-rank test were used for comparing survival in different groups. Endpoints for outcome assessment were PFS, which was defined as the time of surgery to the occurrence of local recurrence or distant metastases, and RCC-specific survival (RCC-SS), which was defined as death from RCC .All analyses were conducted with the SPSS 10.1 software, and *P*-value significance was fixed at 0.05.

## Results

### Patients and tumour characteristics

Sixty-eight (66.7%) and 51 patients (50%) had good performance status and no symptoms at diagnosis, respectively. Tumours were organ-confined (pT1-2) and low-grade (G1-2) in 58 (56.9%) and 37 cases (36.3%), respectively (Table 1). In 12 cases, nodal invasion was present at diagnosis (11.7%) and in 29 cases (28.4%) patients had synchronous distant metastases. During a median follow-up time of 31 months (1–62), 40 patients (39.2%) experienced disease progression and 27 subsequently died from cancer (26.5%).

### Genetic and epigenetic *VHL* gene alteration – relationship with clinicopathological variables and outcome

A *VHL* gene mutation was found in 70 cases (68.6%). Mutations occurred in exon 1–3 in 30 (42.8%), 26 (37.2%) and 14 cases (20%), respectively. Stop, frameshift, missense, splice site and other mutation types were found in 10 (14.3%), 31 (45.7%), 19 (27.1%), six (8.6%) and four cases (4.3%), respectively (Supplementary Table S1). Although there was a trend for better PFS and RCC-SS in patients with *VHL*-mutated tumours, it did not achieve statistical significance (log-rank test, *P*=0.1 and 0.2, respectively). Mutation type and site of mutation did not impact either on PFS or on RCC-SS.

**Figure 1:**
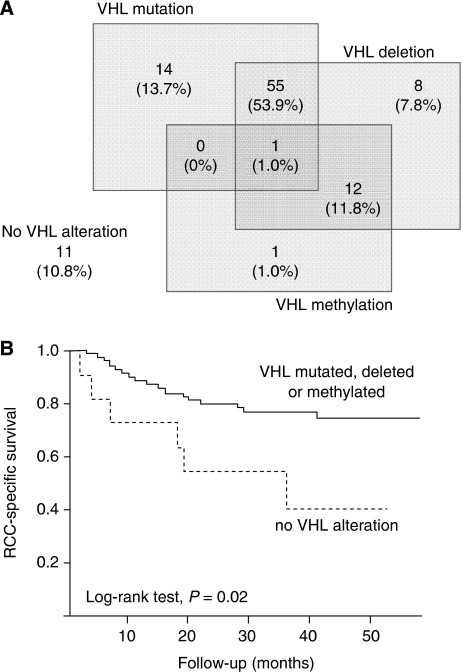
Alteration of the VHL gene in 102 CCRCC tumours. (**A**) Distribution of the different VHL alterations identified. (**B**) Cause-specific survival curve for patients with CCRCC based on VHL alterations (solid line, mutation, deletion or methylation of the VHL gene, *n*=91, 89.2%; broken line, no VHL alteration, *n*=11, 10.8%).

*VHL* deletion and promoter methylation occurred in 76 (74.5%) and 14 cases (13.7%), respectively (Figure 1A). Overall, *VHL* mutation, deletion or hypermethylation occurred in 91 cases (89.2%). When considering patients with no *VHL* mutation, deletion or hyper-methylation (*n*=11, 10.8%), it appeared that their outcome was significantly worse both for PFS and RCC-SS than patients having at least one *VHL* alteration (log-rank test, *P*=0.02; Figure 1B). This group was significantly associated with higher N stages (*P*=0.007) and Fuhrman grades (*P*=0.03).

At least two alterations of the *VHL* gene, potentially leading to biallelic alteration, were found in 68 tumours (66.7%), whereas no or a single alteration of the *VHL* gene was found in 34 patients (33.3%). Comparison of these two groups yielded borderline prognostic significance for PFS (log-rank test, *P*=0.05) but not for RCC-SS (log-rank test, *P*=0.2). There was no relation with tumour size (*P*=0.6), T stage (*P*=0.2), N stage (*P*=0.4) or M stage (*P*=0.7).

### pVHL pattern of expression and relationship with clinicopathological variables

**Figure 2:**
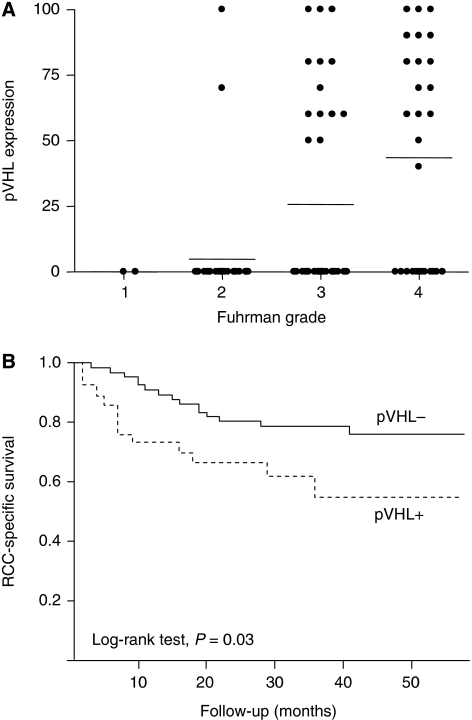
pVHL expression in CCRCC. (**A**) Percentage of tumour tissue with positive pVHL expression according to Fuhrman grade. Horizontal lines represent mean values. (**B**) Cause-specific survival curve for patients based on pVHL expression (solid line, tumours not expressing pVHL, *n*=71, 69.6%; broken line, tumours expressing pVHL, *n*=31, 30.4%).

In normal kidney, a strong cytoplasmic staining was observed in tubular cells as well as a weak staining in mesangial cells. In tumours showing pVHL expression, the staining was strong, diffuse and cytoplasmic. pVHL tumour expression was not detected in 71 cases (69.6%). The absence of pVHL expression was associated with lower N stages (*P*=0.02), M stages (*P*=0.01), Fuhrman grades (*P*=0.001; Figure 2A) along with lower rates of tumour necrosis (*P*=0.005). Finally, absence of pVHL expression was associated with a more favourable outcome measured both by PFS (*P*=0.02) and RCC-SS (log-rank test, *P*=0.03; Figure 2B).

**Figure 3:**
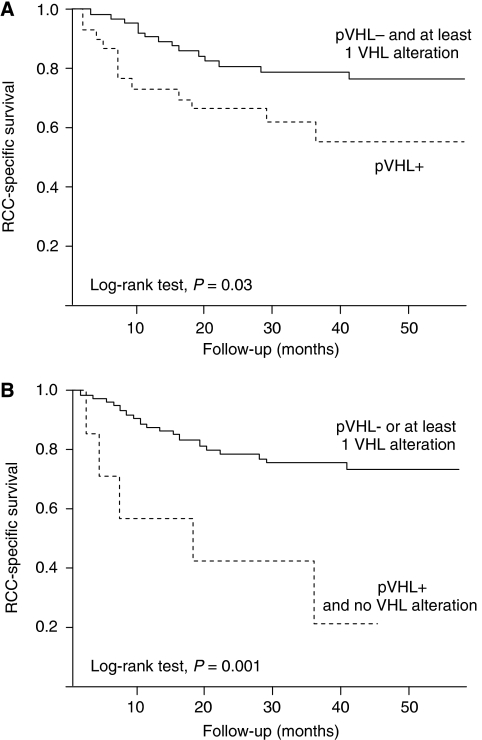
Cause-specific survival curves for patients with CCRCC based on VHL alterations and pVHL expression. (**A**) Survival curves for patients with tumours not expressing pVHL and containing at least one VHL alteration (solid line, *n*=67, 68.4%), and for patients with tumours expressing pVHL (broken line, *n*=31, 31.6%). (**B**) Survival curves for patients with tumours not expressing pVHL or containing at least 1 VHL alteration (solid line, *n*=95, 93.1%), and for patients with tumours expressing pVHL and presenting no VHL alteration (broken line, *n*=7, 6.9%).

When selecting tumours with undetectable pVHL and at least one *VHL* alteration (*n*=67, 68.4%) and comparing this former group to tumours expressing pVHL (*n*=31, 31.6%), the first group exhibited better outcome both in terms of PFS (log-rank test, *P*=0.02) and in terms of RCC-SS (log-rank test *P*=0.03; Figure 3A). A small group of tumours (*n*=7, 6.9%) expressed pVHL and did not present any *VHL* alteration. This group had a median PFS of 7 months compared with 58 months for the rest of the population (log-rank test, *P*=0.004). The median RCC-SS time was 18 months for this particular group with no *VHL* abnormality, whereas it was not reached for the rest of the population (log-rank test *P*=0.001; Figure 3B).

### VEGF tumour and plasma expression – relationship with clinicopathological variables and outcome

**Figure 4:**
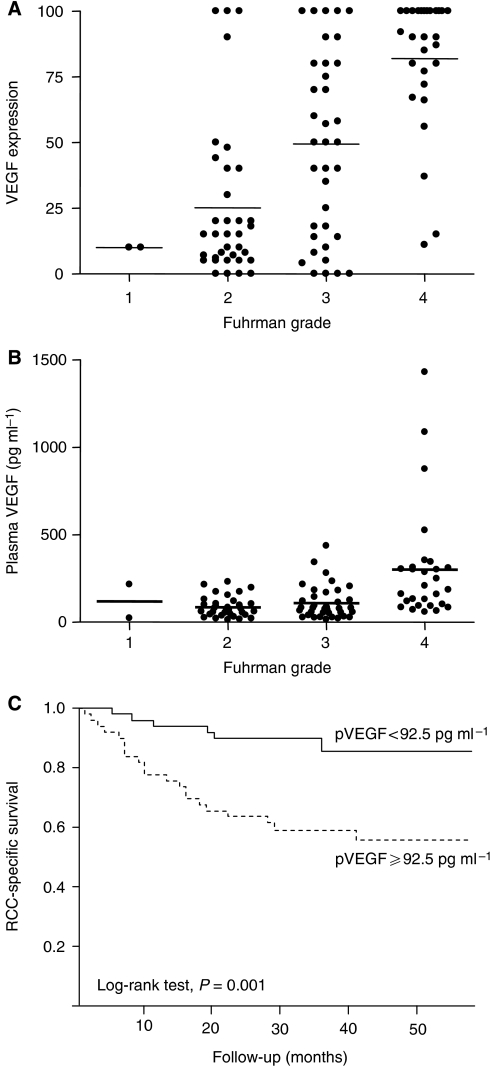
VEGF expression in CCRCC. (**A**) Percentage of tumour tissue with positive VEGF expression according to Fuhrman grade. Horizontal lines represent mean values. (**B**) Concentration of VEGF in the plasma of CCRCC patients according to Fuhrman grade. Horizontal lines represent mean values. (**C**) Cause-specific survival curve for patients based on VEGF expression in plasma (solid line, patients with plasma VEGF<92.5 pg ml^−1^, *n*=56, 50%; broken line, patients with plasma VEGF ⩾92.5 pg ml^−1^, *n*=56, 50%).

Positive staining for VEGF was defined as a membranous and/or cytoplasmic staining pattern of tumour cells. Weak staining was observed in podocytes and endothelial cells in non-tumoral kidney tissue. Median VEGF tumour expression was 49% (0–100). In 60 cases (58.8%) VEGF tumour expression was ⩾30%. The expression of VEGF in tumour was associated with T stage (*P*=0.03), M stage (*P*=0.03), Fuhrman grade (*P*=0.001; Figure 4A) but not with N stage (*P*=0.2) or ECOG performance status (*P*=0.4) (data not shown).

Median VEGF plasma value was 92.5 pg ml^−1^ (13–1430), which is superior to what is reported for healthy donors (Adams *et al*, 2000). Plasma VEGF level was associated with T stage (*P*=0.001), Fuhrman grade (*P*=0.005; Figure 4B), ECOG performance Status (*P*=0.02), but not with N stage (*P*=0.07) or M stage (*P*=0.5; data not shown). Tumour and plasma VEGF were both predictors for PFS (log-rank test, *P*=0.01 and 0.002, respectively) and RCC-SS (log-rank test, *P*=0.05 and 0.001, respectively; Figure 4C). In summary, increased plasma or tumour VEGF expression was associated with more aggressive tumour pattern and poorer outcome.

### Relationship between pVHL, VHL and VEGF

A strong relationship was observed between VEGF tumour expression, VEGF plasma level and pVHL detection (Table 2). However, unexpectedly, the absence of pVHL detection was associated with both lower VEGF tumour and plasma expression (*P*=0.0001 and 0.002, respectively). Similarly, tumours presenting biallelic inactivation of the *VHL* gene were associated with decreased VEGF tumour expression (*P*=0.0001) and plasma VEGF levels (*P*=0.005; Table 2). Consistently, when selecting tumours with no detectable pVHL and at least one *VHL* alteration, it appeared that this latter group exhibited decreased VEGF tumour expression (*P*=0.0001) and VEGF plasma levels (*P*=0.002) compared with tumours expressing pVHL. Finally, the group not harbouring any *VHL* abnormality (*n*=11, 10.8%) was also characterised by increased mean VEGF plasma (309.8±281.9 *vs* 183.4±139.9 pg ml^−1^, *P*=0.03) and mean VEGF tumour expression (77.4±32.4 *vs* 45.8±37.4, *P*=0.009) compared with tumours with either mutation, deletion or methylation of the *VHL* gene (*n*=91, 89.2%).

**Figure 5:**
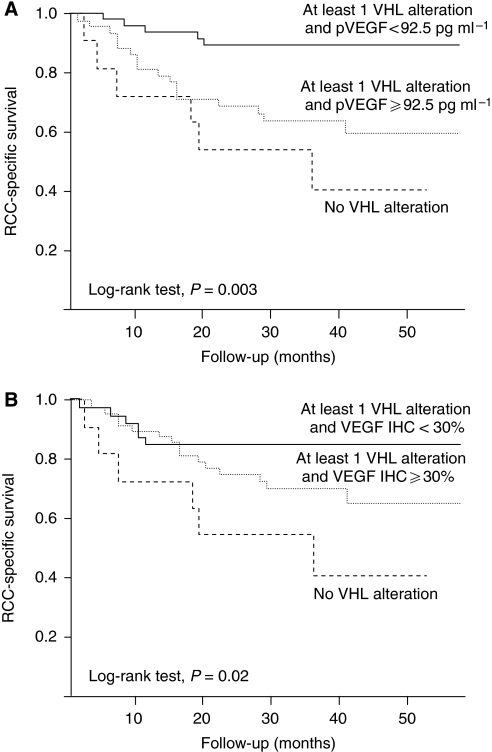
Cause-specific survival curves for patients with CCRCC based on VHL alterations and VEGF expression. (**A**) Survival curves for patients based on VHL alterations and plasma VEGF (solid line, patients with at least one VHL alteration and plasma VEGF<92.5 pg ml^−1^, *n*=48, 47.1%; dotted line, patients with at least one VHL alteration and plasma VEGF ⩾92.5 pg ml^−1^, *n*=43, 42.1% and broken line, patients with a wild-type VHL, *n*=11, 10.8%). (**B**) Survival curves for patients based on VHL alterations and tumour VEGF expression (solid line, patients with at least one VHL alteration and less than 30% of cells expressing VEGF, *n*=40, 39.2%; dotted line, patients with at least one VHL alteration and more than 30% of cells expressing VEGF, *n*=51, 50.0% and broken line, patients with a wild-type VHL, *n*=11, 10.8%).

When focusing on the population with at least one *VHL* alteration, it appeared that both VEGF tumour expression (cut-off 30%) and VEGF plasma level (cut-off, median value of 92.5 pg ml^−1^) were able to stratify two population subsets with distinct outcomes. Based on these finding, our total CCRCC population was, therefore, stratified into three distinct groups: Group 1, at least one *VHL* alteration and low VEGF expression; Group 2, at least one *VHL* alteration and high VEGF expression and Group 3, no *VHL* alteration regardless of VEGF expression. When stratifying with plasma VEGF level, the median PFS times for the three groups were 58, 27 and 17 months, respectively (log-rank test, *P*=0.005), whereas median RCC-SS time was not reached for the first two groups and was 36 months for the third group (log-rank test, *P*=0.003; Figure 5A). When stratifying the entire clear-cell population with VEGF tumour expression, the median PFS times for the three groups were not reached for the first group, was 58 months for the second group and 17 months for the third group (log-rank test, *P*=0.01). In the latest setting, median RCC-SS time was not reached for the first two groups and was 36 months for the third group (log-rank test, *P*=0.02; Figure 5B).

Multivariate analysis has been performed. Unfortunately, it did not reach significance, probably because of the limited number of tumours with no *VHL* alteration.

## Discussion

The importance of tumour angiogenesis in CCRCC development and spreading has been recently strongly validated by the proven efficacy of antiangiogenic drugs that target mainly the VHL/VEGF pathway (Motzer *et al*, 2007; Figlin *et al*, 2008). Up till now, no prognostic factor has been validated in this setting and there is a growing need for defining biological tools for treatment monitoring (Golshayan *et al*, 2008). In this prospective series, we demonstrate that (1) *VHL* alterations appear to be associated with a favourable outcome, (2) VEGF instead of being overexpressed in *VHL*-inactivated tumours seems to be relatively low and (3) a small cohort of CCRCC with no *VHL* alteration exhibit a very unfavourable outcome.

Vascular endothelial growth factor upregulation mechanisms in CCRCC remain hypothetical. It has been demonstrated that VEGF is overexpressed both in tumour tissue and in blood samples in CCRCC (Jacobsen *et al*, 2002; Rioux-Leclercq *et al*, 2007a). It is generally believed that this overexpression is due to *VHL* inactivation that occurs with a high frequency in sporadic CCRCC (70–80%; Gnarra *et al*, 1994; Yao *et al*, 2002). Additionally, it is postulated that *VHL* inactivation leads to HIF constitutive expression and, therefore, induction of target genes including VEGF. However, VHL-independent mechanisms could explain VEGF upregulation and additional role have been described for VHL in CCRCC tumorigenesis (Kim and Kaelin, 2004). Therefore, there was a rationale for analysing in a cohort of sporadic CCRCC the relationship between *VHL* alterations and VEGF plasma and tumour expression.

In this series, we could confirm that plasma or tumour VEGF were elevated in the majority of patients and that both high plasma and tissue VEGF were associated with increased risk of dying from CCRCC. Unexpectedly, mean VEGF tumour expression and mean VEGF plasma levels were significantly increased in tumours with no *VHL* alteration as compared with tumours harbouring at least one *VHL* alteration. This trend was reproducible when examining both pVHL and *VHL* gene. Additionally, out of the 68 tumours with two or three *VHL* alterations, which potentially alter both alleles of the gene, 33 (48.5%) exhibited low VEGF tumour expression, and out of the 34 remaining tumours, 25 (73.5%) had high VEGF tissue expression. It clearly suggests that VHL-independent mechanisms are involved in VEGF upregulation in advanced CCRCC. Conversely, unexpected low VEGF levels were found in tumours with *VHL* alterations, indicating that *VHL* alteration does not necessarily trigger tumour progression.

Using a sensitive approach for mutation detection, we found a 68.6% *VHL* mutational rate along with 74.5 and 13.7% deletion and promoter methylation rates, respectively, which is consistent with what is generally reported in the literature (Banks *et al*, 2006; Nickerson *et al*, 2008). We also demonstrate that VHL abnormalities were associated with a favourable outcome. Our results are in accordance with the report of Yao *et al* (2002) who first presented a correlation between *VHL* mutation and better prognosis. Similarly, Parker *et al* (2005) demonstrated that absence of pVHL detected by immunohistochemistry was associated with improved cancer-specific survival. The present study is also in accordance with our previous analysis focusing on VHL and CAIX (Patard *et al*, 2008b) even though we failed to demonstrate here a clear association between *VHL* gene status and early TNM stages or grades.

Several other studies, however, were not able to correlate *VHL* status to clinicopathological parameters (Kondo *et al*, 2002) or prognosis (Brauch *et al*, 2000; Kim *et al*, 2005; Smits *et al*, 2008), and others linked *VHL* alteration with more advanced tumour stages (Schraml *et al*, 2002). These discrepancies may be partially explained by recruitment bias with an insufficient number of small incidental tumours, or the fact that TNM grouping was used and not separated in T, N and M stage. Methodological bias could also be involved. For example, in most of the studies *VHL* mutation analyses were performed on formalin-fixed, paraffin-embedded material (Schraml *et al*, 2002; Smits *et al*, 2008). It has already been shown for other genes that formalin fixation of archival specimens can induce sequence alterations (Williams *et al*, 1999; Marchetti *et al*, 2006). Finally, the way *VHL* is analysed is also of importance. In most series, tumours with biallelic inactivation of the *VHL* gene are compared with tumours presenting at least one functional allele. When using this approach, we did not find clear correlation with outcome. However, when separating tumours with no *VHL* alteration and tumours with at least one *VHL* hit, we were able to identify two groups non comparable in frequency but with very distinctive clinicopathological features and outcome.

The discrepancy between VHL studies in CCRCC could also be explained by the stage of cancer progression at which VHL status was evaluated. From this point of view, our classification of CCRCC into three different groups with different aggressiveness behaviours according to VHL and VEGF is of interest. The two *VHL*-altered groups with distinct prognostic profiles according to VEGF expression could be reflecting a progression process. VHL alteration, as it is now well admitted, occurs at an early stage of a disease, thus leading to a relative decrease of VEGF expression (Group 1 of our VHL/VEGF classification). These tumours are generally good prognostic tumours that are likely to be cured by surgery or that could be potentially good candidates for immunotherapy in case of metastases. Carbonic Anhydrase IX is generally overexpressed in such tumours and CAIX has been proven to be a good selection criteria for response to interleukin-2-based immunotherapy (Atkins *et al*, 2005; Patard *et al*, 2008b). Tumours with *VHL* alterations and high VEGF levels (Group 2) probably reflect additional molecular events following initial *VHL* alteration. These tumours with VEGF-driven aggressiveness would be good candidates for receiving tyrosine kinase inhibitors (TKIs) that target VEGF-R and PDGF-R. It is probably in this particular group that VEGF should be evaluated in the future as a predictive factor for response to TKIs. Two different studies suggested that tumours with loss of function of *VHL* were more likely to be sensitive to anti-VEGF drugs, thus suggesting that both VHL and VEGF status are potentially important for predicting response to TKIs (Rini *et al*, 2006; Choueiri *et al*, 2008b).

The third group with no *VHL* alteration could be seen either as a distinct molecular entity with no involvement of the VHL pathway. From this point of view it is interesting to note that the survival of patients with poor prognostic metastatic RCC according to the Memorial Sloan-Kettering Hospital Cancer Center (MSKCC) classification is increased by using mTOR inhibitors that do not target specifically the VHL/VEGF pathway (Hudes *et al*, 2007; Motzer *et al*, 2008). It is also interesting to note that non-clear-cell RCCs, which are never *VHL*-mutated, seem to be less sensitive to VEGF targeting drugs than their clear-cell counterpart, and that by contrast non-clear-cell RCCs seem to be sensitive to mTOR inhibitors (Choueiri *et al*, 2008a). Consistent with that vascular phenotype selection hypothesis, metastatic CCRCC after an initial phase of response to TKIs inevitably escape and progress.

In conclusion, classifying CCRCC according to VHL/VEGF status could potentially help physicians to choose the appropriate therapeutic strategy according to the molecular stage of the disease: surgery/immunotherapy, surgery/TKIs and mTOR inhibitors. We investigated a relatively small series of 102 patients with CCRCC. Consequently, subgroups are limited in size. A larger number of tumours with no *VHL* alteration would be needed to validate further our hypotheses through prospective clinical trials, and non-VHL molecular pathways that are involved in CCRCC should be better characterised.

## Figures and Tables

**Table 1:** Summary of the clinical and histopathological characteristics of the 102 patients with sporadic CCRCC

Variables	Value/number
Age (years)	64.5 (21–83)
Sex (male/female)	44 (43.1%)/58 (56.9%)
Tumour size (cm)	7 (1.5–22)
	
*Fuhrman grade*	
1	2 (1.9%)
2	35 (34.3%)
3	37 (36.3%)
4	28 (27.5%)
	
*Tumour stage*	
1	38 (37.3%)
2	20 (19.6%)
3	40 (39.2%)
4	4 (3.9%)
	
*Lymph node status*	
0	90 (88.3%)
1	8 (7.8%)
2	4 (3.9%)
	
*Metastasis status*	
0	73 (71.6%)
1	29 (28.4%)

Abbreviation: CCRCC=clear-cell renal-cell carcinoma.

Pathological diagnosis is according to the Fuhrman grading system and UICC tumour-node-metastasis staging system. Values are presented as median (minimum−maximum) for continuous variables and number of patients (percent) for categorical variables.

**Table 2:** Relationship between *VHL* gene inactivation and VEGF tumour or plasma expression

	2 or 3 *VHL* alteration (*n*=68)	0 or 1 *VHL* alteration (*n*=34)	*P*-value	pVHL− (*n*=71)	pVHL+ (*n*=31)	*P*-value
Mean VEGF tumour expression (%±s.d)	40.3±35.5	68.9±36.5	0.0001	38.7±35.9	73.4±31.6	0.0001
VEGF tumour expression ⩾30%	35 (51.5%)	25 (73.5%)	0.007	33 (46.5%)	27 (87.1%)	0.0001
Mean plasma VEGF level (pg/ml±s.d.)	116.4±90.4	323.2±238.4	0.005	112.9±87.0	326.8±250.2	0.002
VEGF plasma level ⩾92.5 pg/ml	31 (45.6%)	19 (55.9%)	0.2	33 (46.5%)	17 (54.8%)	0.5
Presence of tumour necrosis	34 (50.0%)	17 (50.0%)	0.6	29 (40.8%)	22 (71.0%)	0.005

Abbreviations: pVHL=von Hippel-Lindau protein; s.d.=standard deviation; VEGF=vascular endothelial growth factor; *VHL*=the von Hippel–Lindau gene.

Values are presented as mean±s.d. for continuous variables and number of patients (percent) for categorical variables
