# Supplementary material for: Absence of VHL gene alteration and high VEGF expression are associated with tumour aggressiveness and poor survival of renal-cell carcinoma
Source: Br J Cancer. 2009 Sep 15;101(8):1417–24. doi: 10.1038/sj.bjc.6605298 (PMC2768461; doi:10.1038/sj.bjc.6605298)
Supplement: Supplementary Table S1 legend [file 6605298x2.doc]

Supplementary table S1 legend

“comprehensive tumor, VHL and outcome data in 102 patients with renal clear cell carcinomas”
